# Cross-Cultural Adaptation and Preliminary Reliability of the Adolescents and Adults Coordination Questionnaire into European Spanish

**DOI:** 10.3390/ijerph18126405

**Published:** 2021-06-13

**Authors:** Laura Delgado-Lobete, Rebeca Montes-Montes, David Méndez-Alonso, José Antonio Prieto-Saborit

**Affiliations:** 1 Health Integration and Promotion Research Unit (INTEGRA SAÚDE), Faculty of Health Sciences, University of A Coruña, 15011 A Coruña, Spain; l.delgado@udc.es; 2 TALIONIS Research Group, Research Centre of the Galician University System, Centre for Information and Communications Technology Research (CITIC), Universidade da Coruña, 15008 A Coruña, Spain; 3 Faculty Padre Ossó, University of Oviedo, 33008 Oviedo, Spain; davidm@facultadpadreosso.es (D.M.-A.); josea@facultadpadreosso.es (J.A.P.-S.)

**Keywords:** developmental coordination disorder, health instrument, motor coordination, cross-cultural adaptation, reliability

## Abstract

Developmental Coordination Disorder (DCD) is a developmental disorder affecting motor coordination skills, that frequently persists into adolescence and adulthood. Despite this, very few instruments exist to identify DCD in this population, and none of them are available for Spanish young adults. The purpose of this study was to cross-culturally adapt and preliminarily validate the Adolescents and Adults Coordination Questionnaire (AAC-Q) into European Spanish. The AAC-Q was translated and adapted following international recommendations, including: (a) two independent forward translations; (b) synthesis and reconciliation; (c) expert committee review; and (d) a comprehensibility test. In addition, the internal consistency and homogeneity were examined using a sample of 100 Spanish higher education students. Cultural equivalence and idiomatic differences were addressed to produce the AAC-Q-ES. Findings show that the AAC-Q-ES is a cross-culturally adapted instrument with good preliminary reliability indicators in Spanish young adults (Cronbach’s α = 0.74; corrected item-total correlations = 0.217–0.504).

## 1. Introduction

Developmental Coordination Disorder (DCD) has an estimated prevalence of 5–6% in school-aged children worldwide, but it persists as well during adolescence and adulthood [1]. Previous studies show that adolescents and adults with DCD experience more difficulties in their daily performance, including work and academic activities, social participation, executive functions and organizational skills [2,3,4,5]. In addition, it has been reported that young adults with coordination difficulties have higher prevalence of internal and social problems, poorer physical health and lower feelings of quality of life than their typically developing peers and they internalize these problems [6,7,8,9].

It is important to public health to increase and promote research on DCD in adolescents and young adults, especially in countries such as Spain, where DCD is a highly unknown and underdiagnosed disorder even during childhood [10,11,12]. For that reason, it is very likely that Spanish adults with DCD are unaware of their health condition, which further limits their capacity to seek intervention approaches. In order to get a diagnosis of DCD, the person has to be assessed for the four DSM-5 DCD diagnosis criteria (A = deficits in the motor coordination skills; B = persistent and significant impact of motor deficits in daily living performance; C = early onset of symptoms; D = the motor skills deficits are not better explained by intellectual, developmental, physical or neurological conditions) [13], but it has been repeatedly alerted that the DCD is more underdiagnosed in adolescents and adults [14,15,16]. Therefore, providing accessible, easy-to-use and easy-to-interpretate screening tools may be of great assistance in both clinical and research fields [17,18].

In order to conduct studies on DCD in the Spanish population, health practitioners need cross-culturally adapted screening instruments that are reliable and valid. Some diagnostic measures are currently available that show good cross-cultural equivalence and psychometric properties to evaluate DCD in Spanish children, such as the European Spanish versions of the Developmental Coordination Disorder Questionnaire (DCDQ) and the DCDDaily-Q, which allows for a quick identification and assessment of diagnostic criterion B in school-aged students [19,20,21]. However, these instruments are not appropriate for its use in older population, as criterion B specifically refers to those activities of daily living that are relevant to chronological age. Thus, this criterion needs to be evaluated with caution when assessing for DCD in adolescents and young adults to make sure that the activities that are being measured are culturally and age relevant. During the last decade, several instruments have been designed to contribute to filling this gap, such as the Adults Developmental Coordination Disorder/Dyspraxia Checklist [15], the Adolescents and Adults Coordination Questionnaire (AAC-Q) [16] and the Functional Difficulties Questionnaire-9 (FDQ-9) [14]. All three questionnaires have demonstrated good validity and discriminant capacities to identify adults with DCD, but none of them have been cross-culturally adapted nor validated in further populations or countries. Because of its easy application and interpretation, its good psychometric properties and its brief format, the AAC-Q is an excellent tool to quickly identify adolescents and young adults at risk of DCD and to evaluate diagnostic criterion B in this population. The AAC-Q was originally developed for Israeli young adults, showing excellent internal consistency, test–retest reliability and discriminant validity [16]. The AAC-Q has been used to explore not only the presence of risk of DCD in young adults but also to assist in the research on the daily functioning, quality of life and executive functions in this population as well [2,3,22]. For instance, Tal Saban et al. used the AAC-Q to identify young adults with DCD and to explore their academic and non.academic functioning in cross-sectional and longitudinal studies [2,3]. By providing professionals with economic and reliable instruments, such as the AAC-Q, research on DCD in adolescents and adults may be enhanced, and it could contribute to promoting the research and clinical assessment of DCD in Spain.

Previous studies have demonstrated that DCD-related questionnaires aimed to identify risk of DCD must undergo a rigorous process to ensure its cross-cultural equivalence prior to testing their psychometric properties in the target population in order to verify that the items are culturally relevant, semantically correct and easy to interpretate [19,20,23,24,25,26,27]. Thus, it is necessary to conduct a cross-cultural adaptation of the AAC-Q into European Spanish before performing a comprehensive psychometric validation study and developing reference norms in the Spanish population.

The aims of this study were (a) to cross-culturally adapt the AAC-Q into European Spanish, and (b) to preliminarily test its reliability in Spanish young adults.

## 2. Materials and Methods

### 2.1. The Adolescents and Adults Coordination Questionnaire (AAC-Q)

The AAC-Q is a brief, ecological and self-report questionnaire aimed to identify DCD in adolescents and young adults [16]. It was originally developed to quickly screen risk of DCD based on the daily performance of twelve items reflecting everyday activities, such as self-care activities, instrumental activities, handwriting, social participation or leisure. Each item is rated using a 5-point Likert scale (1 = never or 0% of the time, 2 = occasionally or 25% of the time, 3 = often or 50% of the time, 4 = frequently or 75% of the time and 5 = always or 100% of the time). The final score is obtained by summing the responses of all items, with lower scores indicating better daily motor coordination function. The AAC-Q was validated in Israeli adolescents and young adults, showing excellent internal consistency, test–retest reliability and discriminant validity (Cronbach’s α = 0.90; r = 0.94, *p* < 0.001). In addition, the overall and sex-specific cut-off points of the AAC-Q were calculated using a randomly selected sample of 2379 adolescent and young adult Israeli participants [16].

### 2.2. Ethical Considerations

Authors obtained permission from the original developers of the AAC-Q to cross-culturally adapt and validate this instrument for the Spanish population. Ethical approval for this study was granted by the Ethics Committee of A Coruña-Ferrol (code 2020/535), and all participants gave informed consent to participate in the research following the provision of an information form.

### 2.3. Cross-Cultural Adaptation

**Figure 1:**
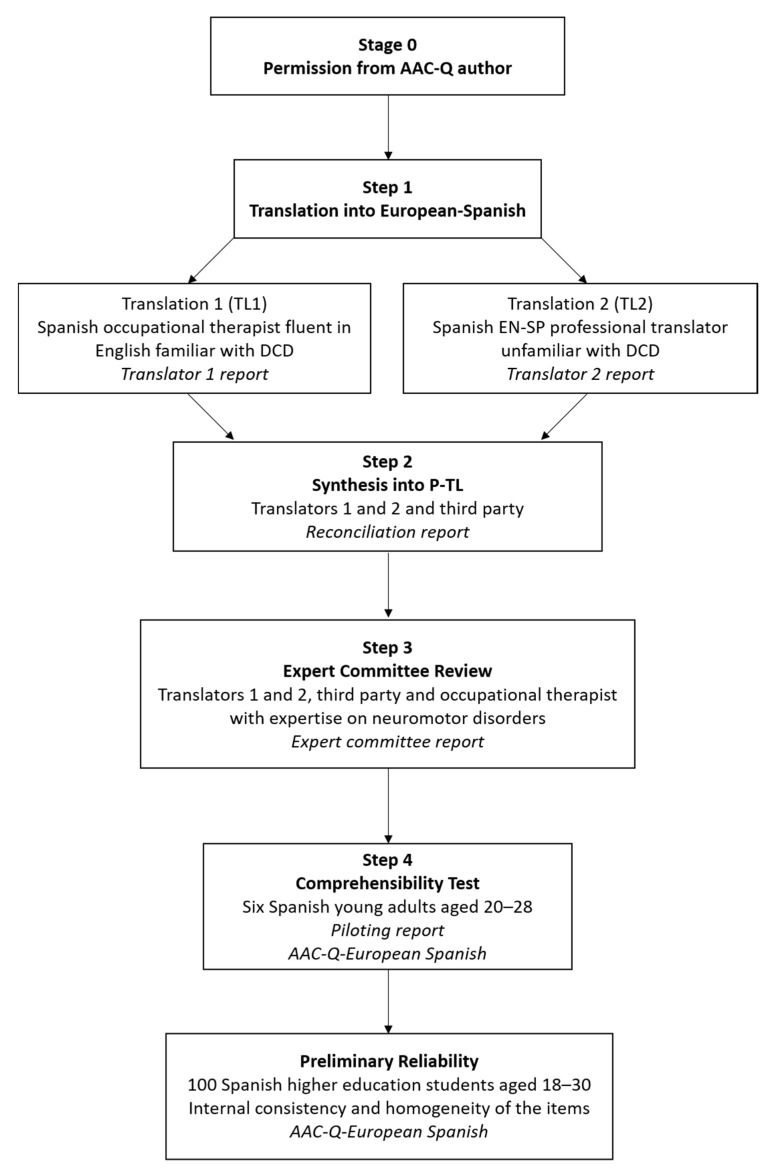
Cross-cultural adaptation process of the European Spanish AAC-Q.

The cross-cultural adaptation of the AAC-Q into European Spanish was conducted following international recommendations. It comprised four steps: forward translation, synthesis, expert committee review and a comprehensibility test (Figure 1) [28,29,30].

#### 2.3.1. Step 1. Two Independent Forward Translations into European Spanish

The English version of the AAC-Q was translated into European Spanish by two independent translators. The first translator was a Spanish occupational therapist fluent in English with research expertise on cross-cultural adaptations of instruments aimed to assess DCD. The second translator was a professional translator unfamiliar with the AAC-Q or DCD. Both translators ranked the difficulty to obtain a translation of the AAC-Q that retained the original meaning of each item from 0 to 10 (low difficulty = 0–3; moderate difficulty = 4–6; high difficulty = 7–10).

#### 2.3.2. Step 2. Reconciliation and Synthesis of the Forward Translations

The instructions, response format and items of the two forward-translated version of the AAC-Q (TL1 and TL2) were compared by the two translators and a third party with previous expertise on cross-cultural adaptation of DCD-related questionnaires. In this step, the ambiguities and discrepancies of both translated versions were discussed and assessed to achieve a consensus and obtain a single, synthesized preliminary translation of the AAC-Q into European Spanish (P-TL). Decisions on the reconciliation of TL1 and TL2 were made based on source and comprehensibility, cultural equivalence, grammatical criteria and terminology [31].

#### 2.3.3. Step 3. Expert Committee Review and Harmonization

A multidisciplinary committee was formed that included the two translators used in Step 1 and Step 2, the researcher that participated in Step 2, and an occupational therapist with expertise in neuromotor disorders in childhood and youth. The four experts were given the original AAC-Q, the TL1, the TL2 and the P-TL. Each aspect of the P-TL (i.e., instructions, response scale and items) were rated either as A (the item is both semantically and conceptually equivalent to the original AAC-Q), B (the item is conceptually equivalent to the original AAC-Q, though minor semantic changes are present) or C (the item cannot be considered equivalent to the original AAC-Q), and potential discrepancies or misunderstandings were further discussed.

#### 2.3.4. Step 4. Comprehensibility Test

A pilot testing was conducted with six higher education students aged 20–28 years, through individual cognitive debriefing interviews (Table A1). This step is recommended to further support the cross-cultural equivalence of the translated questionnaire and to ensure that the items and instructions are easily understood by the target population before assessing its psychometric properties [28,29,30]. Participants were asked to read each question out loud, and then to discuss whether they found it clear and familiar. Finally, they were asked to provide a brief example of each item. If needed, items considered unclear or unfamiliar were further discussed and reworded.

### 2.4. Preliminary Reliability

Preliminary reliability was tested with a sample of University of A Coruña students aged 18–30. Participants were excluded if they: (a) reported a physical, sensory, mental or developmental disability >33%; or (b) reported a clinical diagnosis of a neurodevelopmental disorder, learning disability or health condition affecting movement.

Students enrolled in undergraduate, Master or PhD programs at the University of A Coruña received an online questionnaire on February 2021 that included the information form and informed consent, the European Spanish version of the AAC-Q and a socio-demographic questionnaire to gather information about age, sex, clinical diagnosis of any disability, neurodevelopmental or learning disorders, health conditions affecting movement, student status and the field of study of the participants. Internal consistency and homogeneity of the items were tested with a sample of randomly selected 100 students matched for sex with similar age distribution (females = 50%; mean age males = 22.8, SD = 3.3; mean age females = 22.2, SD = 3.2; *p* = 0.371).

### 2.5. Data Analysis

Data analyses were performed using the IBM Statistical Package for Social Sciences version 26 (SPSS Inc., Chicago, IL, USA). The preliminary reliability of the Spanish version of the AAC-Q was measured with Cronbach’s alpha coefficient, with values above 0.70 indicating an adequate internal consistency. The homogeneity of the items was tested using corrected item-total correlation coefficients, with values above 0.200 being considered acceptable.

## 3. Results

### 3.1. Cross-Cultural Adaptation

Most of items were considered easy to translate into European Spanish by the two translators and did not need a significant conceptual modification in the forward translation (translator 1 = 2.1, SD = 1.1; translator 2 = 0.9, SD = 0.8). Only two items were considered moderately difficult to translate by one translator (i.e., items 6 and 7) (Table 1).

The reconciliation and synthesis process led to a more comprehensive assessment and modification of both translations, which resulted in the agreement of the preliminary translation of the AAC-Q into European Spanish. Each item of the two forward-translated AAC-Q versions was specifically assessed by both translators and the third party to address any discrepancies or potential misunderstandings and ambiguities. Table 2 shows an example of items reconciliation based on previously proposed criteria [31].

In this step, some items underwent further modifications to improve the cross-cultural equivalence of the P-TL. For example, the word “musical” was removed from item 1, since the Spanish expression “*tocar un instrumento*” [“playing a musical instrument”] inherently implies the use of a musical instrument. Other modifications were conducted to make the items sound more natural in European Spanish, such as the change of order of the examples of item 2 (original version: “I tend to be clumsy, fall often, drop items or bump into objects like: closely packed furniture, crowded spaces, narrow passageways”; P-TL: “I tend to be clumsy: I fall often, drop items or bump into objects like: narrow passageways, crowded spaces or closely packed furniture”). In addition, some further examples were included in the P-TL (e.g., using “martial arts (judo, karate, etc.)” instead of “martial arts” in item 4) or further specified (e.g., using “pens, rubbers and pencils” instead of “writing implements” in item 9) to promote a better understanding of the items.

All the items of the P-TL were considered either “both semantically and conceptually equivalent” or “conceptually equivalent” by all four experts, and all items were rated “both semantically and conceptually equivalent” by at least two experts (Table 1), so no further modifications were made during the expert committee review.

Finally, all participants in the comprehensibility test considered the instructions and item wording of the P-TL well written and easy to recall and to answer. The six young adults were able to provide an example for each item that was close to their daily living experience. Only one brief modification in item 3 was made after this step, as two participants proposed using the term “actividades cotidianas” [“*everyday activities*”] instead of “actividades diarias” [“*daily activities*”]. After this last step, the final version of the AAC-Q-European Spanish (AAC-Q-ES) was produced (Table S1 of supplementary material).

### 3.2. Preliminary Reliability

The internal consistency and homogeneity of the items indicated that the AAC-Q-ES has good preliminary reliability for both male and female young adults (overall Cronbach’s α = 0.74) (Table 3). For the overall sample, the α coefficient did not increase when items were individually removed, and only item 12 showed a corrected item-total correlation < 0.200 in the female group.

## 4. Discussion

Research on motor coordination difficulties or DCD in adults is extremely scarce, especially in the Spanish context, even though DCD is a chronic disorder with liming consequences on health and daily functioning, which frequently co-occurs with difficulties in psychosocial health and additional disorders [3,6,9,32]. However, adolescents and adults are very rarely screened for DCD [15,33,34]. The lack of adequately adapted and validated instruments aimed to quickly identify risk of DCD or to assess in the clinical diagnosis may contribute to hinder the diagnosis of this disorder. Thus, it is a priority to provide health practitioners with culturally adjusted, reliable and easy-to-use tools to assess DCD diagnosis. This research work describes the cross-cultural adaptation and preliminary validation of the AAC-Q into European Spanish.

### 4.1. Cross-Cultural Adaptation

The cross-cultural adaptation process led to several minor semantic and conceptual modifications of the translated items, moreover during the first two steps (i.e., forward-translation and reconciliation/synthesis). It must be noted that all four experts agreed that the synthesized P-TL version better expressed the original intended meaning of the items in a way that sounded more natural in European Spanish and that was easier to recall in comparison with the two independent forward translations. This finding contributes to further support that the reconciliation and synthesis is a crucial step in the cross-cultural adaptation process of health-related measures, and that it must not be overlooked [28,29,30,35]. In addition, the value added by including two different translator profiles into the forward translation step was substantial as it allowed us to develop a translation that was both semantically correct and that better reflected the original meaning regarding activities involving motor coordination, organization or challenging behaviors for people with DCD or motor coordination difficulties. Using this combination of expert and professional translators has proved successful in previous cross-cultural adaptations of DCD-related questionnaires in international contexts [19,20,23,27,36,37]. Furthermore, all sections of the P-TL version were considered as both conceptually and semantically equivalent to the original AAC-Q by at least two experts, and all items were considered at least conceptually equivalent in the expert committee review. The cross-cultural equivalence of the European Spanish AAC-Q was finally tested on a sample of Spanish higher education students, which only led to a minor change in one of the items (i.e., item 3, as previously reported). Overall, the AAC-Q-ES is easy to understand, retains good cross-cultural equivalence with the original AAC-Q, and its activities and examples are familiar and close to the daily experience of Spanish young adults.

Conducting a cross-cultural adaptation is advised before using a health-related instrument in a new population that has different language or culture than the population used to originally develop and validate the tool. There are several reasons that further support the specific relevance of this process for those measures aimed to assess daily motor performance. In the first place, these questionnaires are usually self-reported, and in many cases are filled out individually, so it is important that each question is semantically precise and close to the linguistics of the target population in order to ensure the comprehensibility, understanding and good interpretation of the items. For example, the most frequently used tool to assess criterion B in children, the DCDQ [1,38], uses a very specific expression (e.g., “bull in a china shop”) that has experienced several modifications in its international cross-cultural adaptations [20,27,39]. Poorly worded or confusing items could result in unprecise responses, which, as a result, would lead to an inaccurate diagnosis of DCD.

Second, DCD-related items aim to reflect functionality and performance in significant and culturally relevant activities of daily living. Thus, it is necessary to test that the activities that serve as examples are still valid in the target population context. Previous studies have reported the need to adapt some of the activities in DCD-related questionnaires to adjust them to the cultural characteristics (e.g., using “buttering toast” instead of “buttering a sandwich” in the European Spanish version of the DCDDaily-Q, or using “building a playhouse” instead of “building a cardboard or cushion fort” in the European French version of the DCDQ) [19,27]. In this study, only a few minor modifications were made to the original examples of the AAC-Q (e.g., adding “aerobic activities” in item 4), but no substantial changes were needed. The activities and examples of the AAC-Q address a broad range of activities that include not only motor-based activities, but organizational skills, social participation and executive functioning as well, reflecting the many daily areas that are challenging for people with DCD. The findings from this would suggest that these experiences may be relevant across different cultural contexts.

### 4.2. Preliminary Reliability

The examination of the preliminary reliability of the AAC-Q-ES showed good internal consistency and homogeneity in both male and female Spanish young adults. However, the Cronbach’s α of the AAC-Q in the Spanish population was lower to that reported by Tal Saban et al. in the original validation work (Spanish = 0.74, Israeli = 0.88), which could be due to differences in the sample size between both studies (Spanish = 100; Israeli = 2379). To the best of our knowledge, this is the first study to ever evaluate the cross-cultural equivalence or reliability of this instrument in a new population, so it is necessary to replicate the validation studies of the AAC-Q in other populations to test if the good psychometric properties of this instrument are retained across different cultural contexts. The findings from this research contribute to support the use of the AAC-Q in young adults. However, further studies are needed to comprehensively assess the psychometric properties of this measure and to develop specific reference norms for Spanish population before using the AAC-Q-ES to identify Spanish adolescents and adults at risk of DCD, or to evaluate DCD diagnostic criterion B in this population.

### 4.3. Limitations, Strengths and Further Research Directions

This study is subject to several limitations. First, linguistic variety exists in Spain, where different Spanish dialects are spoken in some southern, northeastern and northwestern regions, so this cross-cultural adaptation has been conducted in standard (Castilian) Spanish to promote usability in every Spanish region. In addition, expert committee members and participants in the comprehensibility test came from different regions in order to better address potential differences or confusing aspects of the items. Second, all participants of the reliability study came from a single University. Thus, the comprehensive test of the psychometric properties of the AAC-Q-ES should include young adults from different regions in Spain.

However, this study also has several strengths. As previously noted, translators and members of the expert committee had different professional and cultural backgrounds, which further supports the cross-cultural evaluation of the AAC-Q-ES. In addition, the preliminary reliability test has been examined in a relatively large sample of Spanish young adults, allowing for a sex-specific evaluation.

Overall, the findings from this study show that the AAC-Q-ES is a cross-culturally equivalent instrument with promising reliability values in the Spanish population. A further, comprehensive validity study of the AAC-Q-ES is needed. Once its psychometric properties are confirmed, the AAC-Q-ES can be used to promote DCD research on Spanish adolescents and adults and to assist in the diagnosis assessment of this disorder in the Spanish context.

## 5. Conclusions

The European Spanish version of the AAC-Q is cross-culturally equivalent to the original AAC-Q, and its preliminary reliability results allow for a comprehensive validation test in Spanish young adults. The findings from this study highlight the importance of conducting a rigorous and systematic process when adapting DCD-related instruments in new cultural contexts and populations.

## Figures and Tables

**Table 1:** Translation subjective difficulty and cross-cultural equivalence of the AAC-Q-ES.

AACQ-ES	Translation Subjective Difficulty		Cross-Cultural Equivalence (P-TL)				Reworded in Final Version ^a^
	T1	T2	T1	T2	E3	E4	
Instructions	1	1	A	A	A	A	No
Responses	3	2	A	A	A	A	No
Item 1	2	0	A	A	A	A	No
Item 2	3	1	B	A	A	A	No
Item 3	2	1	B	A	B	A	Yes
Item 4	3	1	B	A	A	B	No
Item 5	1	0	B	A	B	B	No
Item 6	4	3	A	A	A	A	No
Item 7	4	1	A	B	A	A	No
Item 8	1	0	A	A	A	A	No
Item 9	1	1	B	B	A	A	No
Item 10	1	0	A	A	A	A	No
Item 11	2	1	A	A	A	A	No
Item 12	1	1	B	B	A	A	No

AAC-Q-ES = Spanish version of the AAC-Q; T1 = translator 1; T2 = translator 1; E3 = expert 3; E4 = expert 4; P-TL = preliminary translation of the AAC-Q; A = the item is both semantically and conceptually equivalent; B = the item is conceptually equivalent though minor semantic changes are present; ^a^ = reworded after the comprehensibility test.

**Table 2:** Example of items reconciliation and synthesis.

Item 1: I have difficulties with fine motor activities requiring coordinated use of both hands like: threading a needle, cutting, playing a musical instrument, using small tools like pliers, tweezers and screwdrivers, nailing a nail into the wall, replacing a light bulb.	
**TL1**	Me cuesta realizar actividades de motricidad fina que requieren del uso coordinado de las dos manos/ambas manos, como enhebrar una aguja, cortar con tijeras, tocar un instrumento musical, manejar herramientas pequeñas como alicates, pinzas y destornilladores, clavar un clavo en la pared o cambiar una bombilla.
**TL2**	Tengo dificultad con las actividades motoras finas que requieren el uso coordinado de ambas manos como: enhebrar una aguja, cortar, tocar un instrumento musical, utilizar herramientas pequeñas como alicates, pinzas y destornilladores, clavar un clavo en la pared, reemplazar una bombilla.
**P-TL**	Me cuesta realizar actividades de motricidad fina que requieren del uso coordinado de ambas manos, como enhebrar una aguja, cortar con tijeras, tocar un instrumento, manejar herramientas pequeñas como alicates, pinzas y destornilladores, clavar un clavo en la pared o cambiar una bombilla.
**Decision criteria**	Best reflects the stress of the source textReads more naturally in the target languageVocabulary and terminology are consistent throughout the translation

TL1 = translation 1; TL2 = translation 2; P-TL = preliminary translation.

**Table 3:** Internal consistency and homogeneity values of the AAC-Q-ES in overall sample, men and women (*n* = 100).

Items	Total Sample (*n* = 100)		Men (*n* = 50)		Women (*n* = 50)	
	Cronbach’s α if Item Is Deleted ^a^	Corrected Item-Total Correlation	Cronbach’s α if Item Is Deleted ^b^	Corrected Item-Total Correlation	Cronbach’s α if Item Is Deleted ^c^	Corrected Item-Total Correlation
Item 1	0.73	0.299	0.72	0.229	0.72	0.420
Item 2	0.70	0.504	0.67	0.576	0.72	0.398
Item 3	0.72	0.491	0.71	0.331	0.72	0.574
Item 4	0.71	0.488	0.69	0.457	0.71	0.464
Item 5	0.72	0.427	0.72	0.251	0.72	0.471
Item 6	0.73	0.335	0.73	0.260	0.72	0.410
Item 7	0.71	0.471	0.67	0.565	0.73	0.374
Item 8	0.72	0.382	0.70	0.390	0.72	0.383
Item 9	0.72	0.394	0.72	0.224	0.70	0.505
Item 10	0.72	0.384	0.70	0.391	0.73	0.310
Item 11	0.73	0.307	0.68	0.485	0.73	0.267
Item 12	0.74	0.217	0.71	0.344	0.74	0.126

AAC-Q-ES = Spanish version of the AAC-Q; ^a^ = total Cronbach’s α = 0.74; ^b^ = total Cronbach’s α = 0.72; ^c^ = total Cronbach’s α = 0.74.

## Data Availability

The data are not publicly available due to containing information that could compromise the privacy of research participants.

## Supplementary Materials

The following are available online at https://www.mdpi.com/article/10.3390/ijerph18126405/s1, Table S1: European Spanish version of the Adolescents and Adults Coordination Questionnaire (AAC-Q-ES).

## Appendix A

**Table A1:** Sociodemographic characteristics of the comprehensibility test participants.

Participant	Age	Sex	Study Level	Region
1	21	Male	Master student	North
2	28	Female	PhD student	North
3	21	Female	Bachelor student	Northwest
4	23	Female	Bachelor student	Center
5	20	Male	Bachelor student	Northwest
6	25	Male	Master student	Center
