# Supplementary material for: Cross-Cultural Adaptation and Preliminary Reliability of the Adolescents and Adults Coordination Questionnaire into European Spanish
Source: Int J Environ Res Public Health. 2021 Jun 13;18(12):6405. doi: 10.3390/ijerph18126405 (PMC8296233; doi:10.3390/ijerph18126405)
Supplement: Supplementary file 1 [file ijerph-18-06405-s001.zip › ijerph-1262013-supplementary.pdf]

Table S1. The European Spanish version of the Adolescents & Adults Coordination Questionnaire

Cuestionario de Coordinación para Adolescentes y Adultos-Castellano
European Spanish Adolescents & Adults Coordination Questionnaire (AAC-Q-ES)

A efectos aclaratorios, el siguiente cuestionario de autoevaluación proporciona algunos ejemplos junto con cada pregunta. Lee detenidamente las preguntas y los ejemplos antes de responder. En las preguntas en las que solo se incluye un ejemplo, basa tu respuesta en dicho ejemplo.

Por ejemplo:
Ítem nº4: *Tengo dificultades para realizar actividades físicas*.
Por ejemplo: jugar con la pelota, montar en bicicleta, patinar, ir en monopatín, jugar al fútbol, jugar al baloncesto, bailar, actividades de aerobic, deportes extremos, artes marciales (judo, kárate, etc.).

Por favor, marca con una X la respuesta que mejor refleje tu situación en cada pregunta.

Gracias por tu participación

**Ítems**	Nunca (0%)	Algunas veces (25%)	A menudo (50%)	Casi siempre (75%)	Siempre (100%)
Me cuesta realizar actividades de motricidad fina que requieren del uso coordinado de ambas manos, como enhebrar una aguja, cortar con tijeras, tocar un instrumento, manejar herramientas pequeñas como alicates, pinzas y destornilladores, clavar un clavo en la pared o cambiar una bombilla.					
Tiendo a ser torpe: a menudo me caigo, se me caen las cosas de las manos, o suelo tropezar en lugares estrechos, en lugares con mucha aglomeración de gente o donde hay muchos muebles.					
Tengo dificultades en las actividades cotidianas como: abrocharme los botones, atarme los cordones de los zapatos, preparar una comida sencilla, cortarme las uñas, afeitarme, usar una cerilla, maquillarme, planchar, poner en marcha el lavavajillas, ponerme los pendientes, abrochar un collar, hacer el nudo de la corbata.					
Tengo dificultades para realizar actividades físicas como: jugar con la pelota, montar en bicicleta, patinar, ir en monopatín, jugar al fútbol, jugar al baloncesto, bailar, actividades de aerobic, deportes					

extremos, artes marciales (judo, kárate, etc.).					
Tengo dificultades para utilizar herramientas como: un abrelatas, un sacacorchos, un taladro o una sierra eléctrica, una aspiradora, un reproductor de DVD/Blu-ray.					
Tengo dificultades para gestionar mi tiempo, como, por ejemplo: ser puntual, terminar mis tareas, finalizar exámenes u otras actividades dentro del plazo establecido, o planifico mi agenda diaria pero después no termino todo lo que he planeado.					
Me resulta difícil orientarme: me pierdo fácilmente, tengo dificultad para llegar a un sitio nuevo, tengo problemas para reconocer rutas en coche que ya he hecho varias veces o para interpretar un mapa, me cuesta encontrar mi coche en un aparcamiento o garaje, o me cuesta salir de un centro comercial por el mismo sitio por el que he entrado previamente.					
Tengo dificultades para realizar tareas que requieren organización y planificación, como: hacer la maleta antes de un viaje, organizar la ropa en el armario, comprar en el supermercado organizar la cocina, preparar la casa para una celebración o limpiar.					
Suelo perder u olvidar cosas como: bolígrafos, gomas y lápices, abrigos, paraguas, libros, gafas, llaves, teléfono móvil, la cartera.					
Me cuesta aprender nuevos deportes, aunque puedo hacerlos bien una vez que los he aprendido, como por ejemplo nadar, montar en bicicleta, montar en moto o conducir.					
Tengo dificultades para escribir a mano, como: copiar rápidamente de la pizarra, escribir de manera legible, tomar notas durante la clase, o escribo lentamente.					

Me cuesta montar o encajar piezas de forma efectiva y en el orden correcto, como: montar muebles, construir maquetas, hacer puzles o rompecabezas, montar una tienda de campaña.					
